# Combination of smartphone digital image colorimetry and UV-Vis spectrophotometry as detection systems with solidified floating organic drop microextraction as preconcentration method for the quantification of methyl red in wastewater samples

**DOI:** 10.55730/1300-0527.3595

**Published:** 2023-05-31

**Authors:** Miray BOMBOM, Ayça GİRGİN, Buse Tuğba ZAMAN, Fatma TURAK, Sezgin BAKIRDERE

**Affiliations:** 1 Department of Bioengineering, Faculty of Chemistry and Metallurgy, Yıldız Technical University, İstanbul, Turkiye; 2 Department of Chemistry, Faculty of Arts and Science, Yıldız Technical University, İstanbul, Turkiye; 3 Turkish Academy of Sciences (TÜBA), Ankara, Turkiye

**Keywords:** Methyl red, smartphone digital image colorimetry, UV-Vis spectrophotometry, solidified floating organic drop microextraction, textile wastewater

## Abstract

In this study, a portable smartphone-based digital image colorimetric system (SDIC) was designed and integrated with a solidified floating organic drop microextraction method (SFODME) for the quantification of methyl red in textile wastewater samples. The RGB (red, green, and blue) data were evaluated for each captured image, and the green channel was selected for quantification due to its linear response for the analyte. Under optimal conditions, an acceptable linear range was recorded for the analyte. The proposed method recorded a limit of detection (LOD) value of 0.046 mg/L. The developed microextraction method was also combined with UV-Vis spectrophotometry, which recorded an LOD value of 0.012 mg/L. Real sample analysis was carried out with textile wastewater samples to check the applicability/accuracy of the developed method, using a matrix matching calibration strategy to enhance quantification accuracy. Satisfactory percent recoveries in the range of 93.3%–114.3% and 92%–92.7% were recorded for the SFODME-SDIC and SFODME-UV methods, respectively.

## 1. Introduction

Azo dyes are highly stable colored organic compounds within the visible part of the light spectrum, owing to their chromophore parts that contain one or more azo linkages (-N=N-) attached to two or more aromatic rings [1],[2]. They are considered to have excellent biochemical, absorption, nonlinear optic, and emission properties [3],[4]. They have been widely utilized in many fields and products such as fuel cells, thermal transfer printing, molecular photo-switches, dye-sensitized solar cells, and chemo-sensors [5]. However, they can negatively impact both the environment and humans due to their mutagenic, toxic, and carcinogenic features [6]. Additionally, the release of azo dyes into rivers may cause pH changes, hinder the penetration of light through the water body, and cause a reduction in oxygen levels. This has the potential to result in deleterious effects on aquatic organisms and water resources [7]. Methyl red, with the chemical name 2-(4-dimethylaminophenylazo) benzoic acid, is a nonpolar aromatic azo dye that is widely used as a pH indicator in the range of 4.4–6.0 [8] [9]. Besides its use as a pH indicator, it is employed in textile dyeing, ink-jet printing, and laboratory applications [10]. However, it causes negative effects on humans and other organisms [11],[12]. Azo dyes can reduce the absorption of light in the marine environment and generate harmful amine products in the absence of oxygen [13],[14]. For these reasons, accurate quantification of methyl red is very important in evaluating the possible dangers it poses to human health and the environment.

For the determination of azo dyes, high performance liquid chromatography (HPLC) [15], thin-layer chromatography [16], gas chromatography-mass spectrometry (GC–MS) [17], and liquid chromatography tandem mass spectrometry (LC-MS/MS) [18] have been reported in the literature. However, most of these instruments require expert users and their costs are quite high [19]. On the other hand, colorimetric sensors have gained significant attention due to their high sensitivity, simplicity, reliability, and suitability for high-throughput applications [20]. In recent years, colorimetric sensors have been remarkably developed and broadly used to analyze foods such as meat, fruit, seafood, beer and milk, to evaluate their safety for consumption [21].

Recently, smartphone digital image-based colorimetry (SDIC) has received great interest as an analytical technique for both qualitative and quantitative analysis of different samples on account of its advantages such as cost-effectiveness, time-saving analysis, accurate results, simplicity, and portability for on-site applications [22],[23]. Digital image colorimetry is based on the digitalizing of images that are captured by using an acquisition tool, and a design image processing software to obtain the colors in the captured image [24]. The devices employed in obtaining images can be photographic cameras, webcams, smartphones, or scanners [25]. Due to advancements in camera technology, this capacity has been significantly expanded in recent years. Smartphones have the computing power to execute image enhancement tasks without the requirement for specialized hardware or expert human intervention and can also produce reliable and quick results. In addition, data may be simply sent to a network platform for sharing using the mobile phone’s internet connection [26],[27]. During SDIC applications, light that is usually coming from a light-emitting diode (LED) is reflected from the colored sample placed in a colorimetric box and passes through red-green-blue (RGB) filters. The light is then divided into its red-green-blue channels [28], where a linear relationship between the analyte concentration and quantified image data can be established, and the obtained data can be read-out by using applications (APPs) [29]. These APPs are employed for separating the images into their RGB components, which can have values ranging from 0 to 255. Regarding this method, when the intensity of the color increases, the most intense RGB value will proportionally decrease while the other two will slightly increase or stay constant [30]. Due to the advantages of this technique, various analytes such as hydrogen peroxide in milk, and formaldehyde in cosmetic products can be determined quantitatively using a smartphone [31].

In some studies reported in the literature, silica-coated magnetic nanoparticles [32], dispersive liquid-liquid microextraction [33], pipette-tip solid phase extraction [34], silver-nanocomposite-hydrogel-based dispersive solid phase extraction [35], and phase transfer liquid phase microextraction [36] have been used for the extraction/preconcentration of methyl red. Among these extraction methods, DLLME has attracted a lot of attention owing to its advantages, including low cost, high preconcentration efficiency, and fastness [37]. However, the main drawback of DLLME is the use of toxic extraction solvents such as carbon tetrachloride, chlorobenzene, and chloroform [38]. To overcome the disadvantage of DLLME, a new miniaturized liquid phase microextraction, which is called solidified floating organic drop microextraction (SFODME), was first introduced by Zanjani and coworkers in 2006 [39]. The SFODME method involves the use of a few microdrops of low melting point (10–30 °C) organic solvents that settle on the surface of aqueous solution [40]. After adding the microdrops, the sample solution is transferred into an ice bath or freezer until the organic solvent is solidified at the surface of the aqueous solution and taken into a clean tube before instrumental analysis [41]. Advantages of the SFODME method include short extraction time, simplicity, high enrichment factors, minimum consumption of organic solvent, and low cost; thus, it has been widely employed for the determination of both inorganic and organic analytes [42],[43].

The aim of this study was to develop a simple and efficient analytical method for the determination of methyl red. This was achieved by combining a fast, high-sample throughput, and low-cost smartphone digital image colorimetric system and UV/Vis spectrometry with an eco-friendly solidified floating organic drop microextraction method. The microextraction method was applied to yield highly accurate and precise results in the determination of methyl red. The SDIC method was fast, accurate, and user-friendly, making it a viable alternative to advanced analytical systems that are more complex and require specialized expertise. The applicability and accuracy of the analytical method were tested on textile wastewater as a real sample matrix.

## 2. Experimental

### 2.1. Instrumentation

**Figure 1:**
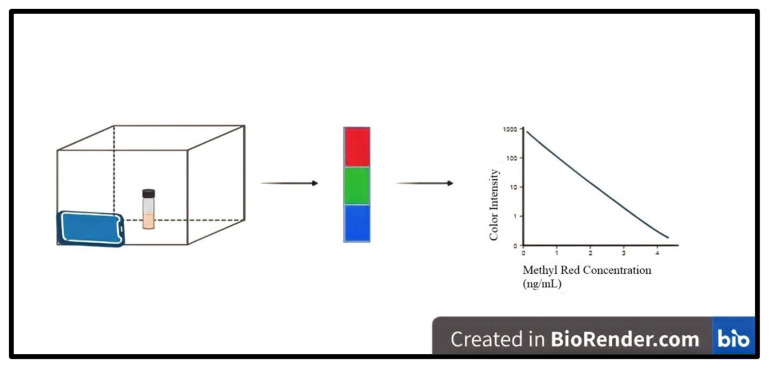
Schematic presentation of SDIC method showing the measurement of a sample solution through the three color channels and the corresponding chart.

Images were captured using an iPhone 7 plus (iOS 7.0) camera with 12-megapixel picture quality and an f1/8 aperture. A rectangular measurement unit, which was made from Styrofoam (20 × 25 cm), was covered with black tape in order to minimize the entrance of external light, and the images were captured through a small hole positioned at one side of the box to acquire high-throughput images. An LED strip (12 V) was placed at the top part of the inner box and the led strip was covered with A4 white paper for the distribution of the radiation emitted inside the box. Another A4 white paper was placed at the bottom of the box and the images were taken with the sample vial placed on it. Measurement vials were placed at a constant position in the middle of the box to allow homogenous light and achieve high repeatability for replicate measurements. The distance between the smartphone fixed on the box cover and the sample vial was determined as 4.50 cm. The light intensities of the captured images were obtained using the Color Detector application, and the calibration plots for each color channel were generated using the averaged intensities from the digital images. Intensity measurements were done taken for the green channel because it yielded linear responses for varying concentrations, whereas the red and blue channels were nonlinear. A schematic diagram of the experimental setup, designed using the BioRender application is illustrated in Figure 1.

UV-Visible measurements were taken with a Shimadzu UV2600 model UV-Vis spectrophotometer (Shimadzu International Trading Co., Ltd., Shanghai, China). Quartz microcells with 2.0 mm internal width and 0.70 mL volume were used as sample holders in the absorbance measurement. The absorbance measurements were performed in the range of 300–900 nm at 1.0 nm intervals.

### 2.2. Chemicals and reagents

Analytical grade chemicals and reagents were used throughout the study. A 1000-mg/L stock standard solution of methyl red was prepared by dissolving its pure solid form (Merck KGaA, Darmstadt–Germany) in ethanol. The calibration standard solutions used for the SDIC measurements were prepared by diluting the stock methyl red solution with ethanol. Ultrapure deionized water was obtained from a Milli-Qâ Reference Ultrapure Water Purification System and used as a diluent for the preparation of aqueous working/calibration standard solutions (Isolab, Germany). Hydrochloric acid (0.10 M) and potassium hydrogen phthalate solution (0.10 M) were mixed together to prepare a pH 4.0 buffer solution. Textile wastewater samples obtained from a textile manufacturing factory were used in the recovery experiments. Potassium hydrogen phthalate, hydrochloric acid, dodecanol, 1-decanol, undecanol, ethanol, and methanol were all acquired from Merck KGaA, Darmstadt – Germany.

### 2.3. Extraction procedure

**Figure 2:**
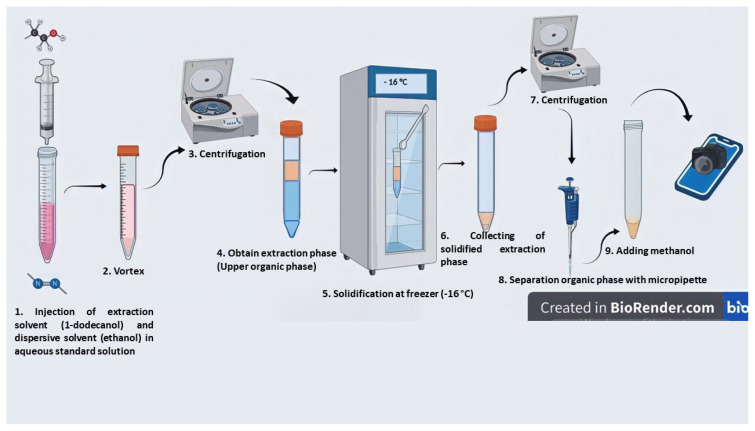
Schematic diagram of the analytical method depicting the experimental steps of extraction and device measurements.

The analytical procedure of the proposed SFODME-SDIC method was carried out in 15-mL centrifuge tubes. The optimum pH condition for methyl red was adapted from the study performed by Atsever et al., where the optimum pH and volume of the buffer solution were determined to be 4.0 and 1.0 mL, respectively [44]. In this procedure, 8.0 mL of aqueous standard solution or sample and 1.0 mL of pH 4.0 buffer solution were pipetted into the 15-mL centrifuge tube. Next, 250 μL of dodecanol (extractant) and 1.50 mL of ethanol (disperser solvent) were mixed in a clean tube, withdrawn and injected via a syringe into the sample solution. The mixture was vortexed for 15 s in order to enhance homogenous distribution of the extraction solvent throughout the sample solution. Subsequently, centrifugation was performed at 3000 rpm for 2.0 min to facilitate the separation of the extraction solvent from the aqueous solution. In order to solidify the upper organic phase, the tube was kept in a freezer at −16 °C. After the solid extraction phase had been collected into a clean tube using a precooled microspatula, 100 μL of methanol was added to reduce the viscosity of the extraction solvent. Finally, light intensity measurements were done by the SDIC and UV-Vis systems. A schematic diagram of the extraction process developed with the BioRender application is depicted in Figure 2.

### 2.4. Real sample preparation

The textile wastewater used as a real sample in this study was taken from the wastewater discharge line of a textile production facility in Türkiye. The collected wastewater samples were kept in a cool and moisture-free environment at +4.0 °C before being used in the study. The sample was covered with aluminum foil to prevent exposure to sunlight. Before preparing the sample solutions for the study, the pH of the wastewater sample was measured and recorded as 7.41. Next, the wastewater sample was centrifuged at 2800 × *g* for 10 min to facilitate settling of solid particles and/or sediments in the sample. The resulting supernatant was filtered through a filter paper with a pore size of 11 μm. Afterwards, appropriate dilutions were made and spiking experiments were performed.

## 3. Results and discussion

All variables with significant effects on the extraction efficiency, including type/volume of disperser solvent, and type/volume of extraction solvent were optimized in order to maximize extraction outputs. Optimization experiments were carried out by using the “one-variable-at-a-time” approach with three replicates. All optimization experiments (Figure S1) with the exception of the extraction solvent selection were performed with the SDIC system. Extraction solvent type optimization was performed using the UV-VİS instrument. The parameter variable with the highest absorbance and a relatively low standard deviation was selected as the optimum one. In the SDIC system, color intensity decreases with the optimization experiments; hence, the lowest color intensity with a lower standard deviation was selected as the optimum channel.

### 3.1. Effect of type and volume of extraction solvent

In the SFODME method, the extraction solvent must have a lower density than water with a low melting point (in the range of 10–30 °C) and be immiscible in water. For this reason, 3 different nonpolar extraction solvents, including 1-decanol (density: 0.8297 g/mL, melting point: 6.4 °C), dodecanol (density: 0.8309 g/mL, melting point: 24 °C), and undecanol (density: 0.8298 g/mL, melting point: 19 °C) were tested under equivalent conditions. Although the analytical signals of 1-decanol and dodecanol were very close to each other, it was observed that phase separation and solidification of the organic phase were more efficient and rapid for dodecanol. Therefore, dodecanol was selected for further optimization.

The volume of the extraction solvent plays a significant role in the extraction process as it can lead to the dilution of extracted analytes when high volumes are used. Contrarily, when low volumes are used, it might be insufficient to collect all the analytes from the solution. In this regard, 150, 200, 250, and 300 mL volumes of dodecanol were tested and 250 mL was chosen as the optimum one (Figure S1).

### 3.2. Effect of type and volume of disperser solvent

To achieve higher extraction recovery, a disperser solvent can be used to increase the surface area between the aqueous solution and the extraction solvent. With the addition of a disperser solvent, a homogenous distribution of the extraction solvent throughout the aqueous solution occurs. Thus, three disperser solvent types (methanol, 2-propanol, and ethanol) were tested to select the optimum one. It was determined that there was no statistically significant difference between the results obtained for the solvent types tested. Ethanol was selected as the optimum disperser solvent for the subsequent experiments.

After determining the disperser solvent type, the effect of its volume on extraction efficiency was examined. Changes in the upper organic phase volume, size of the organic drops, and polarity of the aqueous phase could affect the yield of the microextraction method; thus, it is essential to evaluate the disperser solvent volume. For this reason, ethanol volumes of 1.0, 1.5, and 2.0 mL were tested. There was no statistical difference observed between the results. In addition, it was observed that 1.0 mL of ethanol was not sufficient to effectively disperse the extraction solvent throughout the solution, and thus uniform turbidity did not occur. Furthermore, it was observed that the solubility of the analytes in water increased with increasing volume of the disperser solvent, specifically for the disperser volume of 2.0 mL. This led to an incomplete extraction procedure, as evidenced by the absence of phase separation. As a result of all these observations, the optimum volume of the disperser solvent was selected as 1.5 mL (Figure S1).

### 3.3. Analytical figures of merit

Under the optimum experimental conditions presented in Table 1, a calibration plot was developed using the average green color intensities of triplicate measurements for methyl red standard solutions. Linearity was evaluated for the three color channels (red, green, and blue) and linearity was observed for the green color channel. Each replicate was captured three times, and three points were recorded to obtain average RGB values for each image. In this regard, smartphone digital image colorimetry was used, and the results were read out via the Color Detector application to evaluate the calibration plots for the determination of analytical figures of merit. The colorimetric method showed a linear dynamic range of 0.10–0.50 mg/L with an R^2^ value of 0.9890. The lowest concentration at which the signal-to-noise ratio is equal to or greater than 3 (n = 6) and the slope of the calibration plot were used to compute the limit of detection (LOD). The limit of detection value was obtained by dividing three times the standard deviation by the slope, and the limit of quantification (LOQ) value was determined using 10/3 times. LOD was obtained as 0.046 mg/L, and other analytical parameters of the developed system are presented in Table 2.

The analytical performance of the developed extraction method was also examined by measuring the standard/sample solutions in a UV-Vis spectrophotometry system besides the digital image-based colorimetry system. The main purpose was to use another detection system for the quantitative determination of the analyte and compare it to the developed analytical method.

**Figure 3:**
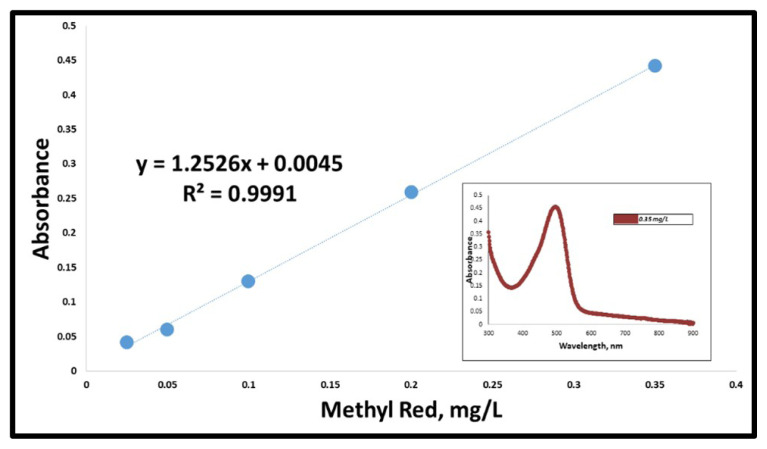
Calibration plot obtained for methyl red in UV-Vis spectrometry system and a corresponding absorbance signal for 0.35 mg/L standard solution.

Aqueous methyl red solutions with increasing concentrations were prepared and the optimum extraction procedure was applied to these solutions. The obtained extract solutions were then scanned in the wavelength range of 300–900 nm. According to the results, identification/quantification of methyl red was performed at 497 nm by virtue of the highest absorbance value being recorded at this wavelength. The UV spectra obtained in the linear dynamic range of the analyte and the calibration plot developed for the results of the UV spectrophotometry system are depicted in Figure 3.

**Figure 4:**
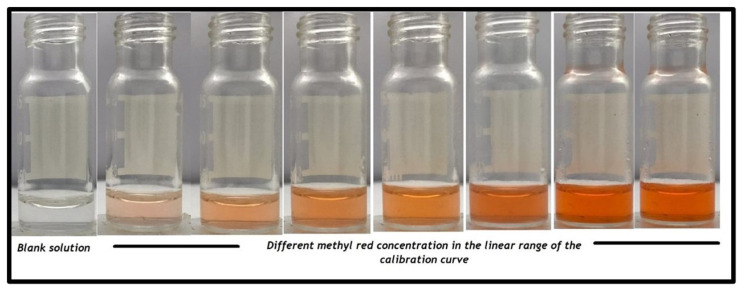
Photographs depict the different concentrations of methyl red in linear range of the developed SFDOME-SDIC method.

The LOD value was calculated using the calibration plot displayed above and the value was recorded as 0.012 mg/L with a linear dynamic range of 0.025–0.35 mg/L. Photographs taken for different concentrations of the analyte under the optimum conditions can be seen in Figure 4.

### 3.4. Recovery studies

The applicability of a new analytical method to be used for quantitative determinations may be adversely affected by the components of a sample matrix. Therefore, spike recovery experiments were performed on textile wastewater samples in order to verify the applicability and accuracy of the developed SFODME-SDIC method under the determined optimum conditions. The textile wastewater samples were filtered and diluted properly with ultrapure deionized water. Next, 3 replicate blank solutions were prepared to determine whether or not the matrix contained the analyte, but no analytical signals were observed within the detection limit of the method. Subsequently, 0.20, 0.35, 0.50, 0.75, and 1.0 mg/L concentrations were prepared in textile wastewater samples by spiking with proper amounts of the stock standard solution. The spiked textile wastewater samples were then extracted under the optimum conditions and run with aqueous calibration standards in the colorimetric system. Recovery results were obtained using the matrix matching method in order to overcome/minimize possible positive or negative interferences that could affect the analytical signals. As seen in Table 3, the percent recoveries of 0.35, 0.50, and 0.75 mg/L spiked wastewater samples were calculated as 114.3 ± 2.7%, 97.5 ± 2.5%, and 93.3 ± 2.5%, respectively. The accuracy of the results in wastewater samples was improved by applying the matrix-matching strategy. The same experiments were performed in the UV-Vis spectrophotometric system in order to verify the system’s capability to perform quantitative determination of the analyte in a complex matrix. The recovery results recorded for the matrix matching method are also given in Table 3. The percent recovery results obtained between 92% and 93% validated the accuracy and applicability of the developed extraction procedure coupled with the UV-Vis detection system. The high percent recovery values with low standard deviations for both detection systems indicate that the combination of these simple preconcentration and detection systems is a viable alternative to other instrumental methods used for the determination of methyl red in complex matrices.

### 3.5. Comparison with other methods reported for the determination of methyl red

In the study reported by Khodadoust and Ghaedi, methyl red was determined by UV-Vis spectrophotometry after its preconcentration using a dispersive liquid phase microextraction method. The optimum experimental conditions were all determined by multivariate optimization steps, and under these conditions, an LOD value of 5.0 mg/L was recorded [33]. In another study, methyl red was preconcentrated using molecularly imprinted polymer pipette-tip solid phase extraction (MIP-PT-SPE). In the extraction procedure, 2.0 mg of MIP was used for 50 μL of sample solution adjusted at pH 3.0. Under the optimum conditions, a wide linear working range between 0.003 and 0.3 mg/L was obtained, and the LOD was calculated as 0.0005 mg/L [34]. In 2017, Bardajee and coworkers synthesized silver nanocomposite hydrogels by microwave-irradiation-assisted synthesis method to use as adsorbent in the preconcentration of methyl red by dispersive solid phase extraction. The detection limit of the study was reported as 1.4 mg/L, and the developed method showed linearity in the concentration range of 0.1–25 mg/L [35].

## 4. Conclusion

An eco-friendly solidified floating organic drop microextraction method was combined with two detection systems namely smartphone digital image colorimetry (SFODME-SDIC) and UV-Vis (SFODME-UV), for the determination of methyl red. This combination increased the applicability of the methods to complex matrices such as wastewater samples. All the parameters that affect the analytical performance of the extraction method were optimized to achieve high sensitivity with low detection limits. The RGB values were recorded from the obtained images, and the best analytical signals were given in the average green intensities. The limits of detection for the SDIC and UV-Vis systems were calculated as 0.046 and 0.012 mg/L, respectively. The analyte recorded good linearity in both detection systems. Spiked recovery experiments performed on textile wastewater samples validated the applicability of the developed methods to real samples. In order to eliminate matrix effects, the matrix matching method was implemented, and the percent recovery results were recorded in the range of 92%–115% for both SDIC and UV-Vis systems.

## Supplementary Information

Figure S1

## Figures and Tables

**Table 1:** Optimized experimental parameters of the SFODME-SDIC method.

Parameters	Value
**Color channel**	Green
**Extraction solvent type, volum**e	Dodecanol, 250 mL
**Dispersive solvent type, volume**	Ethanol, 1.5 mL
**Vortex period**	15 s
**Solidification temperature, period**	−16 °C, 10 min

**Table 2:** Summarization of the analytical performance values of the method and comparison of these values with other studies in literature.

Method	LOD, mg/L	Linear range, mg/L	Reference
**SFODME SDIC**	0.046	0.10–0.50	This study
**SFODME-UV-Vis Spectrometry**	0.012	0.025–0.35	This study
**DLLME-UV-vis Spectrometry** **a**	5.0	15–10,000	[33]
**MIP-PT-SPE-UV-vis Spectrometry** **b**	0.0005	0.003–0.30	[34]
**SNH-MW-DSPE-UV-Vis spectrophotometer** **c**	1.4	0.1–25	[35]

a Dispersive liquid-liquid microextraction UV-Vis spectrophotometry;

b molecularly imprinted polymer pipette-tip solid phase extraction UV-Vis spectrophotometry;

c microwave-assisted synthesis of a silver nanocomposite hydrogel-dispersive solid phase extraction UV-Vis spectrophotometry.

**Table 3:** Obtained percent recoveries of spiked textile wastewater samples for both analytical systems.

Analytical method	Spiked concentration, mg/L	Found concentration, mg/L	Recovery (%)
**SFODME-SDIC**	0.35	0.40	114.3 ± 2.7
	0.50	0.49	97.5 ± 2.5
	0.75	0.70	93.3 ± 2.5
**SFODME–UV-Vis spectrometry**	0.20	0.18	92.0 ± 5.6
	0.35	0.32	92.7 ± 2.9

## Data Availability

Data will be available on reasonable request.
