# Supplementary figures and images for: Combination of smartphone digital image colorimetry and UV-Vis spectrophotometry as detection systems with solidified floating organic drop microextraction as preconcentration method for the quantification of methyl red in wastewater samples
Source: Turk J Chem. 2023 May 31;47(5):1075–84. doi: 10.55730/1300-0527.3595 (PMC10760811; doi:10.55730/1300-0527.3595)

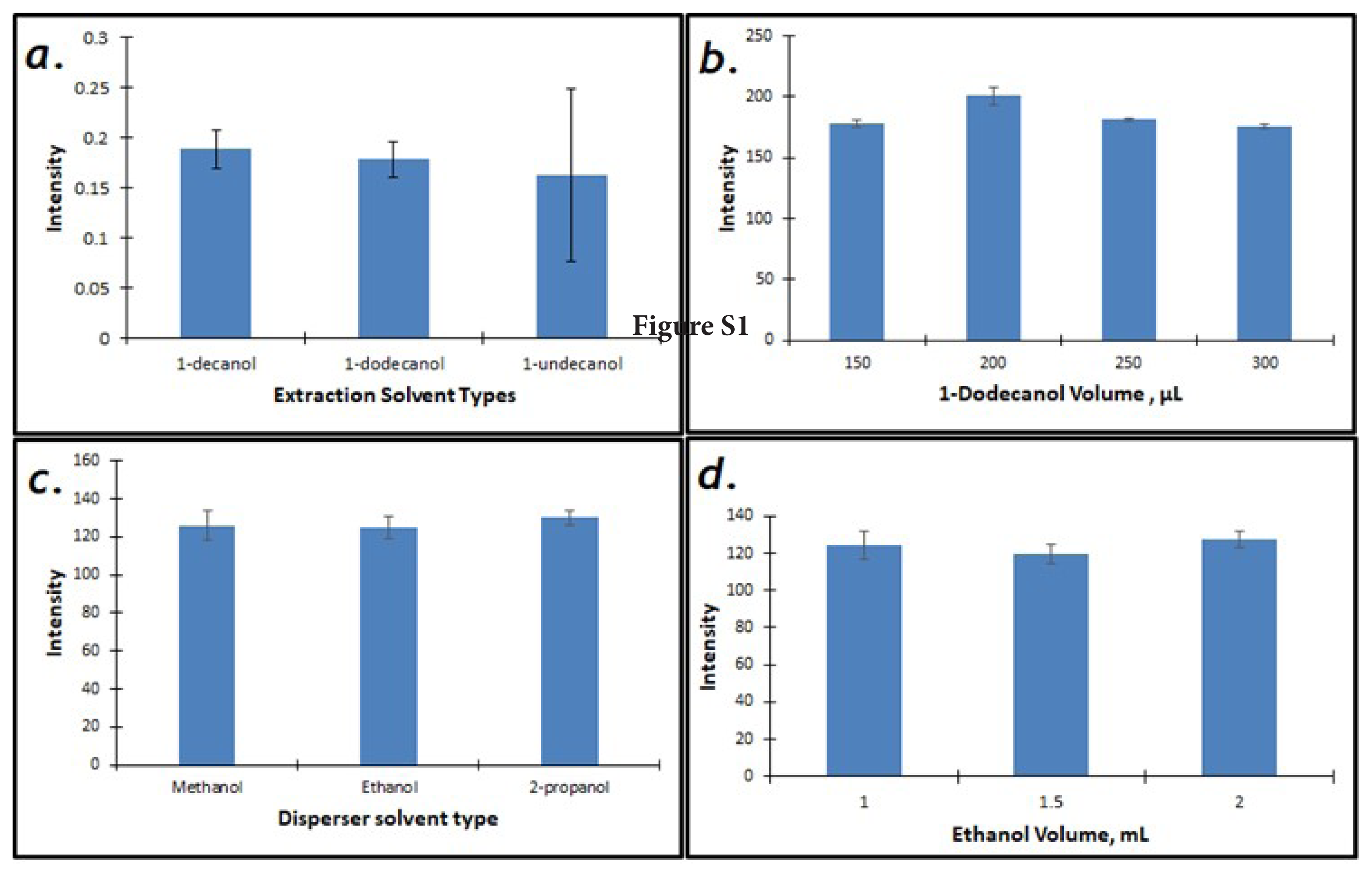

Supplement: Figure S1 [file turkjchem-47-5-1075s1.tif]
